# Effects of the presence of ColE1 plasmid DNA in *Escherichia coli *on the host cell metabolism

**DOI:** 10.1186/1475-2859-5-34

**Published:** 2006-11-17

**Authors:** Zhijun Wang, Li Xiang, Junjie Shao, Alicja Węgrzyn, Grzegorz Węgrzyn

**Affiliations:** 1 Key Laboratory of Medical Molecular Virology, Shanghai Medical College, Fudan University, 200032, Shanghai, People's Republic of China; 2 Department of Population Medicine and Diagnostic Sciences, College of Veterinary Medicine, Cornell University, Ithaca, 14853, NY, USA; 3 Laboratory of Molecular Biology (affiliated with the University of Gdańsk), Institute of Biochemistry and Biophysics, Polish Academy of Sciences, Kładki 24, 80-822 Gdańsk, Poland; 4 Department of Molecular Biology, University of Gdańsk, Kładki 24, 80-822 Gdańsk, Poland; 5 Department of Genetics and Marine Biotechnology, Institute of Oceanology, Polish Academy of Sciences, Św. Wojciecha 5, 81-347 Gdynia, Poland

## Abstract

**Background:**

Although understanding of physiological interactions between plasmid DNA and its host is important for vector design and host optimization in many biotechnological applications, to our knowledge, global studies on plasmid-host interactions have not been performed to date even for well-characterized plasmids.

**Results:**

*Escherichia coli *cells, either devoid of plasmid DNA or bearing plasmid pOri1 (with a single ColE1 replication origin) or plasmid pOri2 (with double ColE1 replication origins), were cultured in a chemostat. We used a combination of metabolic flux analysis, DNA microarray and enzyme activity analysis methods to explore differences in the metabolism between these strains. We found that the presence of plasmids significantly influenced various metabolic pathways in the host cells, e.g. glycolysis, the tricarboxylic acid (TCA) cycle and the pentose phosphate (PP) pathway. Expression of *rpiA*, a gene coding for ribose-5-phosphate isomerase A, was considerably decreased in *E. coli *carrying a high copy number plasmid relative to *E. coli *carrying a low copy number plasmid and plasmid-free *E. coli*. The *rpiA *gene was cloned into an expression vector to construct plasmid pETrpiA. Following induction of pETrpiA-bearing *E. coli*, which harbored either pOri1 or pOri2, with isopropyl-β-D-thiogalactopyranoside (IPTG), the copy number of pOri1 and pOri2 was sigificantly higher than that measured in a host devoid of pETrpiA.

**Conclusion:**

The presence of plasmids can significantly influence some metabolic pathways in the host cell. We believe that the results of detailed metabolic analysis may be useful in optimizing host strains, vectors and cultivation conditions for various biotechnological purposes.

## Background

Plasmids are among the most widely used model replicons and tools in molecular biology and biotechnology. However, only a few reports were published to-date about the effects of plasmid DNA on the metabolism of *Escherichia coli *[1-3]. On the other hand, an optimization of plasmid vectors and host strains has received an important interest in biotechnology, especially due to findng novel applications of plasmids, like gene therapy and development of DNA vaccines [1,4,5]. Undoubtedly, understanding physiological interactions between plasmid DNA and its host is important for vector design and host optimization [1,3,6,7].

The presence of plasmid DNA can have various impacts on the host physiology, including perturbation in DNA replication, transcription and translation [8]. Effects of plasmids on the *E. coli *metabolism were investigated, and it was found that the presence of plasmid DNA caused an increase in glucose uptake rate, and revealed faster drop of the extracellular and intracellular pH and higher accumulation of lactic, acetic, formic, and succinic acids [9]. A hypothesis was presented that plasmids influence host metabolism through changes in the cAMP-binding protein (cAMP)-CRP complex, which in turn causes the substantial alteration in the regulatory status of the glucose uptake rate [8]. Effects of plasmid DNA on the growth rate of host cells were also reported. For example it was found that the growth rate of *E. coli *strain DH1 containing plasmid pGSK001 decreased from 0.63 h^-1 ^to 0.55 h^-1 ^[6], and similar conclusions on the effects of plasmids on bacterial growth were reported by other authors [8].

Birnbaum and Bailey demonstrated that the presence of plasmids in *E. coli *HB101 induced an increase in levels of enzymes involved in the tricarboxylic acids cycle, ribosome assembly, protein biosynthesis and the heat shock response. Moreover, they observed that PEP carboxylase and succinate dehydrogenase were among the proteins whose levels were higher in the presence of plasmids [10]. Rozkov et al. [6] has proposed that plasmids can influence cell metabolism through increasing ATP synthesis, necessary for expression of an antibiotic-resistance gene.

In spite of some work pefromed previously (see above), to our knowledge, global studies on plasmid-host interactions have not been performed to-date even for well-characterized plasmids. It encouraged us to analyse the impact of plasmid DNA on the metabolism of *E. coli *host using methods of the ^13^C flux technology, DNA microarray and enzyme activity analysis.

Metabolic flux analysis is important in characterizing cellular phenotypes [11]. The proteinogenic amino acids serve as valuable probes to study glycolysis, metabolism of pyruvate, the pentose phosphate (PP) pathway and the tricarboxylic acid (TCA) cycle. Mixtures of [U-^13^C]glucose and unlabelled glucose are useful to resolve fluxes downstream of PEP. An exclusive use of [1-^13^C]glucose is valuable for resolving the upper part of the metabolism, close to the PP pathway. Direct analytical interpretation of ^13^C-labelling patterns has been used sucessfully in biochemical research. More recently, analytical interpretation of the ^13^C-labelling pattern in proteinogenic amino acids was developed, and several flux partition ratios can be quantified in a single experiment [12,13]. Net fluxes through metabolic networks can be obtained from ^13^C-labelling information, when combined with material balance within a stoichiometric model [12,14,15]. DNA microarrays have been intensively used to explore the response of wild-type *E. coli *gene expression to a variety of environmental conditions, for example, acetate [16], oxygen availability [17], biofilm formation [18], and protein overproduction [19-21]. Furthermore, enzyme activity analysis has been considered an important method to analyse the metabolism of microorganism [22,23].

Because ColE1-like plasmids belong to the best characterized replicons and the most intensively used plasmids in biotechnology, such replicons were selected as models to investigate plasmid effects on the *E. coli *metabolism.

## Results

### Bacterial growth

**Figure 1:**
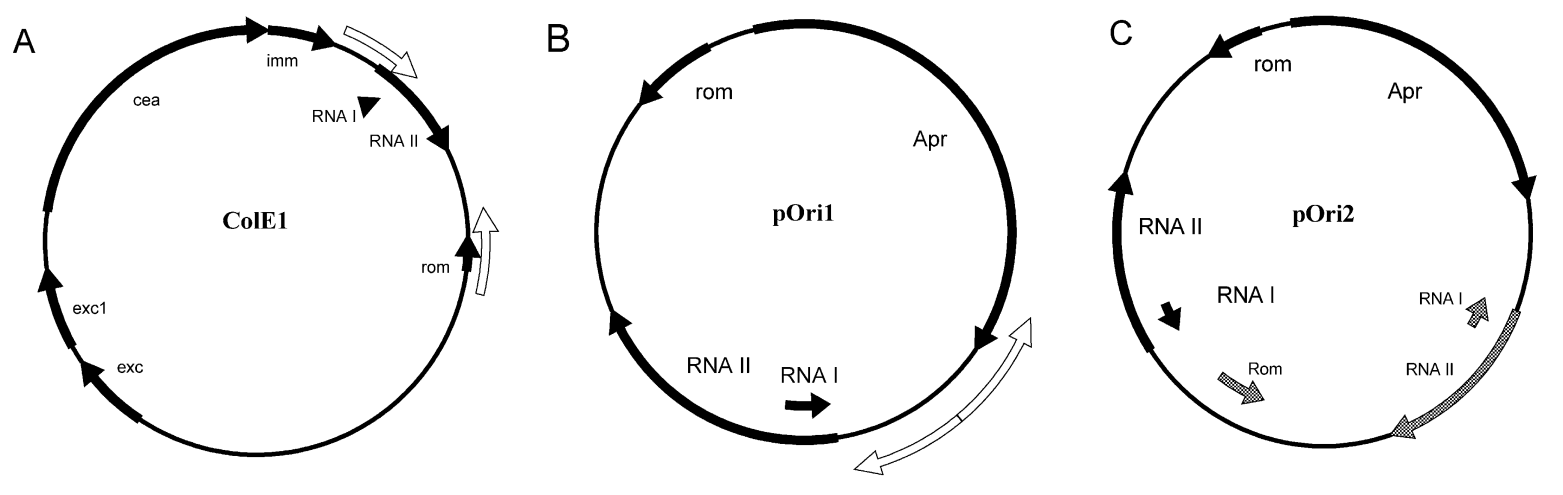
Construction of two ColE1-derived plasmids, pOri1 (2,811 bp) and pOri2 (4,575 bp). The construction was performed as follows: (A): ColE1 replication origin was amplified from ColE1 plasmid DNA with the indicated primers (white arrow in Fig. 1A). The PCR amplified origin fragment was used for construction of pOri1; (B): The PCR product from step A was linked with an ampicillin-resistance gene to construct a new plasmid pOri1. The whole pOri1 plasmid DNA was amplified with the indicated primer (white arrow in Fig. 1B). The PCR amplified whole pOri1 was used for the construction of pOri2; (C): The PCR amplified whole pOri1 product was linked with ColE1 replication origin fragment from step A again to construct a new plasmid pOri2.

Two ColE1-derived plasmids were constructed, which bear either a single ColE1 origin region (pOri1) or two such regions (pOri2). This pair of plasmids was used, instead of other well-characterized low- and high-copy number plasmids, to avoid any unpredictable effects of metabolic differences caused by various types of replicons (the only considerable difference between pOri1 and pOri2 is the number of replication *origin*s; see Fig. 1). To analyse the metabolic differences between *E. coli *strains BL21, BL21/pOri1 and BL21/pOri2, batch cultures were performed in 5 l fermentor BIOSTAT^® ^B-DCU with the working volume of 2 l. The growth rates of BL21, BL21/pOri1 and BL21/pOri2 were compared during bath cultivation at 16 h in the early stationary phase. The growth rates of these strains were 0.46 h^-1^, 0.39 h^-1 ^and 0.29 h^-1^, respectively. The growth rate of BL21 carrying a high copy number plasmid pOri2 was significantly lower than that of BL21 carrying a low copy number plasmid pOri1. These results are compatible with previous observations.

Although low copy number plasmids have a minor contribution to total cellular biomass, DNA amount of high copy number plasmids in a host cell may be considerable in comparison to chromosomal DNA. Thus, one migh speculate that plasmids may cause significant effects on the growth characteristics of *E. coli*. To analyse effects of plasmid DNA on the metabolism of *E. coli*, the chemostat cultures were performed to obtain steady state cultivation under of the growth rates of 0.46 h^-1 ^for BL21, 0.39 h^-1 ^for BL21/pOri1 and 0.29 h^-1 ^for BL21/pOri2, with feeding a complete culture medium. The growth rates were identical to those measured when these three strains were cultured for 16 h in the batch cultivation. The growth characteristics of *E. coli *are shown in Table 1. Plasmid pOri1 copy number in BL21 was estimated to about 50, however the plasmid pOri2 copy number in this strain was about 400. As pOri1 and pOri2 are a pair of otherwise identical plasmids bearing one or two *origin *regions, respectively, we suggest that the significantly increased plasmid DNA copy number in BL21/pOri2 was probably caused by the presence of two *origin*s of replication in this plasmid.

**Table 1:** Growth parameters of *E. coli *strains BL21, BL21/pOri1 and BL21/pOri2 cultured in the chemostat system at different growth rates.

Parameter^a^	Value		
	
	BL21	BL21/pOri1	BL21/pOri2
Q_Gluc_	10.96	35.00	36.01
μ	0.46	0.39	0.29
q_Gluc_	5.04	13.65	10.46
Y_biomass_	0.97	1.95	1.66
y_biomass_	0.45	0.76	0.48
r_Gluc_	5.2	7.0	6.3
r_Ace_	0	4.1	4.4
r_O2_	11.0	16.9	16.5
r_CO2_	12.2	17.9	17.3
Plas. copies	0	50	410

^a ^The following growth rates were determined when bacteria were cultured for 16 h in the batch cultivation: BL21, 0.46 h^-1^; BL21/pOri1, 0.39 h^-1^; BL21/pOri2, 0.29 h^-1^. Abbreviations: Q_Gluc _(mmol), Glucose consumption amount; μ (h^-1^), growth rate; q_Gluc _(mmol h^-1^), Glucose consumption rate; Y_biomass _(g), Biomass yield; y_biomass _(g h^-1^), Biomass yield rate; r_Gluc _(mmol g^-1 ^h^-1^), Glucose consumption rate per biomass; r_Ace _(mmol g^-1 ^h^-1^), Acetate yield per biomass; r_O2 _(mmol g^-1 ^h^-1^), Oxygen uptake rates per biomass; r_CO2 _(mmol g^-1 ^h^-1^), CO_2 _secretion per biomass; Plas. copies, Plasmid copy number

The expenditure of glucose in BL21, BL21/pOri1 and BL21/pOri2 was 5.2, 7.0 and 6.3 mmol g^-1 ^h^-1^, respectively (Table 1). Surprisingly, the expenditure of glucose in BL21/pOri2 was lower than that in BL21/pOri1. Thus, the presence of plasmid DNA pOri1 can significantly improve the glucose expenditure relative to plasmid-free *E. coli *BL21, however the BL21 carrying a high copy number plasmid pOri2 does not increase the expenditure of glucose in comparison to BL21 carrying a low copy number plasmid pOri1. This might be due to the lower early stationary phase growth rate of BL21/pOri2 than BL21/pOri1.

Acetate secretion was low in BL21, however significantly higher acetate secretion was found in BL21/pOri1 (4.1 mmol g^-1 ^h^-1^) and BL21/pOri2 (4.4 mmol g^-1 ^h^-1^) (Table 1). Oxygen uptake rates were increased in plasmid-containing strains over those of the plasmid-free strain (Table 1). Our results indicated that *E. coli *BL21 carrying a high copy number plasmid pOri2 did not increase the oxygen uptake relative to *E. coli *BL21 carrying a low copy number plasmid pOri1. Similar correlations were obtained when CO_2 _secretion was measured (Table 1). In summary, the high copy number plasmid pOri2 in *E. coli *BL21 induced lower growth rates, and higher acetate secretion than the low copy number plasmid pOri1. The presence of plasmid pOri1 or pOri2 can significantly decrease the growth rate, and increase the O_2 _and glucose uptake rates relative to plasmid-free *E. coli *BL21.

Although we have controlled the growth rate of *E. coli*, it has been found that growth rates can affect the growth characteristic of *E. coli *[24]. To analyse the effects of ColE1-like plasmids on the growth characteristics of *E. coli*, strains BL21, BL21/pOri1 and BL21/pOri2 were cultured at the same growth rate (0.2 h^-1^) in the chemostat system. The growth characteristics are shown in Table 2. The expenditure of glucose in BL21, BL21/pOri1 and BL21/pOri2 was 2.6, 4.8 and 5.7 mmol (g biomass)^-1 ^h^-1^, respectively. The Biomass yield rate of BL21, BL21/pOri1 and BL21/pOri2 was 0.161 g h^-1^, 0.261 g h^-1 ^and 0.207 g h^-1^, respectively. However, the plasmid DNA copy number in BL21, BL21/pOri1 and BL21/pOri2 (Table 2) did not change significantly relatively to experiments shown in Table 1.

**Table 2:** Growth parameters of *E. coli *strains BL21, BL21/pOri1 and BL21/pOri2 cultured in the chemostat system at the same growth rate.

Parameter^a^	Value		
	
	BL21	BL21/pOri1	BL21/pOri2
Q_Gluc_	10.4	24.8	29.45
μ	0.20	0.20	0.20
q_Gluc_	2.08	4.96	5.89
Y_biomass_	0.805	1.305	1.035
y_biomass_	0.161	0.261	0.207
r_Gluc_	2.6	4.8	5.7
r_Ace_	0.5	2.4	5.5
r_O2_	7.3	13.1	13.8
r_CO2_	7.7	13.8	14.4
Plas. copies	0	80	420

^a ^The growth rate of each culture was 0.2 h^-1^. The abbreviations are as described in Table 1.

### Enzyme activity analysis

Some key enzymes of central metabolism were assayed during the early stationary phase and at the same growth rate. Activities of 26 enzymes involved in the central metabolic pathways of *E. coli *BL21, BL21/pOri1, BL21/pOri2 during the early stationary phase and at the same growth rate are shown in Table 3 and Table 4.

Activities of the glycolysis pathway enzymes, such as: HEK, GPI, PFK, FBP, FBA, GPD, TPI, PPC and PK, were increased in BL21/pOri1 and BL21/pOri2 relative to BL21 at the early stationary phase and at the same growth rate (Tables 3 and 4). There were no significant differences in activities of glycolysis enzymes' activities between BL21/pOri1 and BL21/pOri2 at the early stationary phase, although there were some increases of HEK, PFK, FBP, FBA, GPD and PCC activities at the same growth rate (Table 4). In summary, our results show that *E. coli *BL21 containing a high copy number plasmid pOri2 does not reveal significantly increased glycolysis enzymes' activities in comparison to the *E. coli *BL21 containing a low copy number plasmid pOri1 (Tables 3 and 4).

**Table 3:** Specific enzyme activities in *E. coli *strains BL21, BL21/pOri1 and BL21/pOri2 cultured in the chemostat system at different growth rates.

Enzyme^a^	Activity (μmol min^-1 ^mg^-1^)^b,c^		
	
	BL21	BL21/pOri1	BL21/pOri2
HEK	0.030	0.100	0.110
GPI	2.290	4.660	4.630
PFK	0.240	0.640	0.600
FBP	0.014	0.043	0.049
FBA	1.210	2.960	2.810
GPD	0.026	0.051	0.052
TPI	1.700	4.400	4.410
PPC	0.220	0.430	0.440
PK	0.044	0.098	0.102
GPDH	0.350	0.120	0.070
PGL	0.280	0.210	0.120
PGD	0.150	0.090	0.080
RPE	0.320	0.150	0.100
RPI	0.540	0.230	0.030
TK	0.080	0.010	0.005
TA	0.120	0.070	0.020
CS	0.021	0.086	0.082
ICD	0.150	0.840	0.830
ICL	0.013	0.046	0.045
AH	0.090	0.190	0.210
OGD	0.022	0.088	0.810
SD	0.020	0.090	0.110
FH	0.021	0.097	0.110
MD	0.046	0.094	0.101
MAC	0.002	0.004	0.003
PCK	0.002	0.003	0.003

^a ^Abbreviations: HEK, Hexokinase; GPI, Glucose-6-phosphate isomerase; PFK, 6-phosphofructosekinase; FBP, Fructose-1,6-bisphosphatase; FBA, Fructose-bisphosphate aldolase; GPD, Glyceraldehyde-3-phosphate dehydrogenase; TPI, Triose phosphate isomerase; PPC, Phosphoenolpyruvate carboxylase; PK, Pyruvate kinase; GPDH, Glucose-6-phosphate dehydrogenase; PGL, 6-Phosphogluconolactonase; PGD, Phosphogluconate dehydrogenase; RPE, Ribulose-phosphate 3-epimerase; RPI, Ribose-5-phosphate isomerase; TK, Transketolase; TA, Transaldolase; CS, Citrate synthase; ICD, NADP^+^-specific isocitrate dehydrogenase; ICL, Isocitrate lyase; AH, Aconitate hydratase; OGD, Oxoglutarate dehydrogenase; SD, Succinic semialdehyde dehydrogenase; FH, Fumarate hydratase; MD, Malate dehydrogenase; MAC, Malic enzyme; PCK, Phosphoenolpyruvate carboxykinase.

^b ^The following growth rates were determined when bacteria were cultured in the batch cultivation: BL21, 0.46 h^-1^; BL21/pOri1, 0.39 h^-1^; BL21/pOri2, 0.29 h^-1^.

^c ^The experiments were performed in triplicate and the results are shown as the average of three measurements with the standard deviation less than 5%.

**Table 4:** Specific enzyme activities in BL21, BL21/pOri1 and BL21/pOri2 strains grown in the chemostat system at the same growth rate.

Enzyme^a^	Activity (μmol min^-1 ^mg^-1^)^b^		
	BL21	BL21/pOri1	BL21/pOri2
HEK	0.012	0.051	0.074
GPI	0.947	2.228	2.085
PFK	0.104	0.241	0.311
FBP	0.004	0.020	0.034
FBA	0.402	1.439	1.937
GPD	0.011	0.023	0.034
TPI	0.719	1.991	2.072
PPC	0.093	0.129	0.253
PK	0.017	0.047	0.043
GPDH	0.108	0.061	0.036
PGL	0.103	0.090	0.058
PGD	0.038	0.040	0.043
RPE	0.107	0.073	0.059
RPI	0.180	0.096	0.013
TK	0.033	0.003	0.003
TA	0.037	0.035	0.014
CS	0.007	0.040	0.055
ICD	0.064	0.361	0.404
ICL	0.005	0.022	0.021
AH	0.030	0.085	0.097
OGD	0.006	0.031	0.369
SD	0.006	0.044	0.067
FH	0.008	0.035	0.069
MD	0.018	0.043	0.065
MAC	0.001	0.001	0.002
PCK	0.001	0.001	0.002

^a ^Abbreviations are as described in the footnote to Table 3.

^b ^The growth rate of each culture was 0.2 h^-1^. The experiments were performed in triplicate and the results are shown as the average of three measurements with the standard deviation less than 5%.

PP pathway enzymes, such as: GPDH, PGL, PGD, RPE, RPI, TK and TA were down-regulated in the BL21/pOri1 or BL21/pOri2 relative to BL21 at the early stationary phase and the same growth rate (Tables 3 and 4), and the PP pathway enzyme activities of BL21/pOri2 were lower than those of BL21/pOri1 (Tables 3 and 4). RPI was the most significantly down-regulated enzyme in the PP pathway in BL21/pOri2 at the early stationary phase and the same growth rate (Tables 3 and 4). Our results showed that *E. coli *BL21 carrying a high copy number plasmid pOri2 revealed lower PP pathway enzyme activities than *E. coli *BL21 carrying a low copy number plasmid pOri1 at the early stationary phase and the same growth rate (Tables 3 and 4).

Tricarboxylic acid (TCA) cycle enzymes, such as: CS, ICD, ICL, AH, OGD, SD, FH and MD revealed significantly increased activities in BL21/pOri1 and BL21/pOri2 relative to BL21 at the early stationary phase (Table 3) and at the same growth rate (Table 4). There were no significant differences in the TCA enzyme activities between BL21/pOri1 and BL21/pOri2 during the early stationary phase (Table 3). These results are in correlation with the higher glucose use and oxygen uptake rate of the BL21/pOri1 and BL21/pOri2 than BL21 (Table 1). Moreover, there were no significant differences in the MAC and PCK enzyme activities among BL21, BL21/pOri1 and BL21/pOri2 (Table 3 and 4).

### Metabolic flux analysis

The metabolic fluxes of BL21/pOri1 and BL21/pOri2 at early stationary phase and the same growth rate were directly compared with those of BL21. We found that protein and lipid contents of BL21/pOri2 (μ = 0.29 h^-1^) and BL21/pOri1 (μ = 0.39 h^-1^) were lower than those of BL21 (μ = 0.46 h^-1^), however the RNA contents of BL21/pOri2 and BL21/pOri1 were higher than those of BL21 at the early stationary phase (Table 5). The cellular ingredients of protein, lipid, RNA, and LPS in BL21, BL21/pOri1 and BL21/pOri2 were determined as constant at the same growth rate (μ = 0.2 h^-1^) except plasmid DNA components. Table 6 shows the ingredients of protein, DNA and RNA in BL21, BL21/pOri1 and BL21/pOri2 at different growth rates. Ingredients of amino acids, nucleotides in BL21, BL21/pOri1 and BL21/pOri2 were different during the early stationary phase, however, the ingredients of amino acids and nucleotides were constant at the same growth rate (Table 6).

**Table 5:** Cellular components of *E. coli *strains BL21, BL21/pOri1 and BL21/pOri2 cultured in the chemostat system at different growth rates.

Component	Biomass g (g biomass)^-1^			
	Various growth rates^a^			0.2 h^-1^
		
	BL21	BL21/pOri1	BL21/pOri2	All strains

Protein	0.620	0.615	0.611	0.610
Lipid	0.119	0.109	0.102	0.102
RNA	0.185	0.200	0.205	0.211
Chromosomal DNA	0.031	0.031	0.031	0.031
Plasmid	0	0.0002	0.0019	^b^
LPS	0.015	0.015	0.015	0.015
Peptidoglycan	0.015	0.015	0.015	0.015
Glycogen	0.015	0.015	0.015	0.015

^a ^The following growth rates were determined when bacteria were cultured in the batch cultivation: BL21, 0.46 h^-1^; BL21/pOri1, 0.39 h^-1^; BL21/pOri2, 0.29 h^-1^.

^b ^Plasmid DNA concentrations in BL21, BL21/pOri1, and BL21/pOri2 were 0, 0.0004, and 0.0020 g (g biomass)^-1^, respectively.

**Table 6:** Levels of amino acids and nucleotides in *E. coli *strains BL21, BL21/pOri1 and BL21/pOri2 cultured in the chemostat system at different growth rates.

	Biomass (mmol g^-1^)			
Component	Various growth rates^a^			0.2 h^-1^
		
	BL21	BL21/pOri1	BL21/pOri2	All strains
	
Ala	0.940	0.976	0.995	0.996
Arg	0.465	0.511	0.529	0.531
Asp	0.620	0.465	0.438	0.43
Asn	0.465	0.474	0.474	0.474
Cys	0.146	0.146	0.146	0.146
Glu	0.520	0.529	0.538	0.539
Gln	0.566	0.438	0.374	0.373
Gly	0.849	0.411	0.319	0.318
His	0.146	0.164	0.173	0.175
Ile	0.438	0.447	0.474	0.476
Leu	0.547	0.575	0.630	0.63
Lys	0.538	0.566	0.566	0.567
Met	0.237	0.328	0.356	0.357
Phe	0.310	0.401	0.411	0.412
Pro	0.347	0.438	0.438	0.438
Ser	0.438	0.529	0.529	0.539
Thr	0.493	0.575	0.575	0.576
Trp	0.109	0.128	0.164	0.165
Tyr	0.255	0.347	0.319	0.32
Val	0.693	0.675	0.675	0.661
dATP	0.782	0.782	0.782	0.782
dTTP	0.782	0.782	0.782	0.782
dCTP	0.807	0.807	0.807	0.807
dGTP	0.807	0.807	0.807	0.807
ATP	0.807	0.807	0.807	0.807
UTP	0.665	0.665	0.665	0.665
CTP	0.616	0.616	0.616	0.616
GTP	0.992	0.992	0.992	0.992

^a ^The following growth rates were determined when bacteria were cultured in the batch cultivation: BL21, 0.46 h^-1^; BL21/pOri1, 0.39 h^-1^; BL21/pOri2, 0.29 h^-1^.

**Figure 2:**
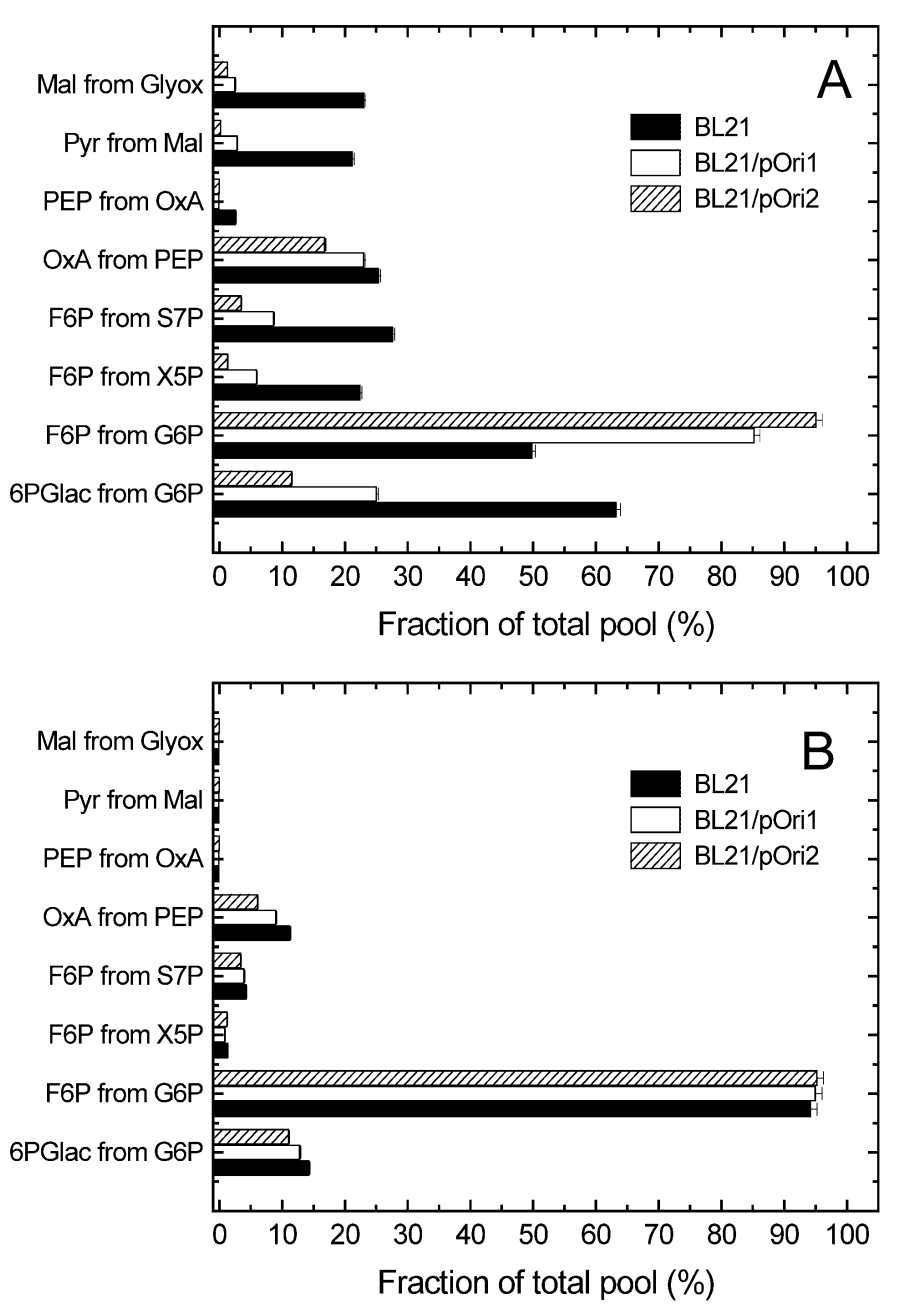
Analysis of metabolic intermediates during cultivation of *E. coli *BL21 (0.46 h^-1^), BL21/pOri1 (0.39 h^-1^) and BL21/pOri2 (0.29 h^-1^) at the early stationary phase in a chemostat-fermentor system (A), and cultivation of *E. coli *BL21 (0.2 h^-1^), BL21/pOri1 (0.2 h^-1^) and BL21/pOri2 (0.2 h^-1^) at the same growth rate (B).

To determine the metabolic fluxes of *E. coli *carrying different copy number plasmids, we performed the ^13^C glucose labelled experiments, in which the metabolic ratios of several metabolic nods were determined. The metabolic flux ratios of intracellular metabolites of BL21, BL21/pOri1 and BL21/pOri2 were analysed with the FiatFlux 1.0 software [25]. The metabolic flux ratio results at different growth rate are shown in Fig. 2A and 2B.

**Figure 3:**
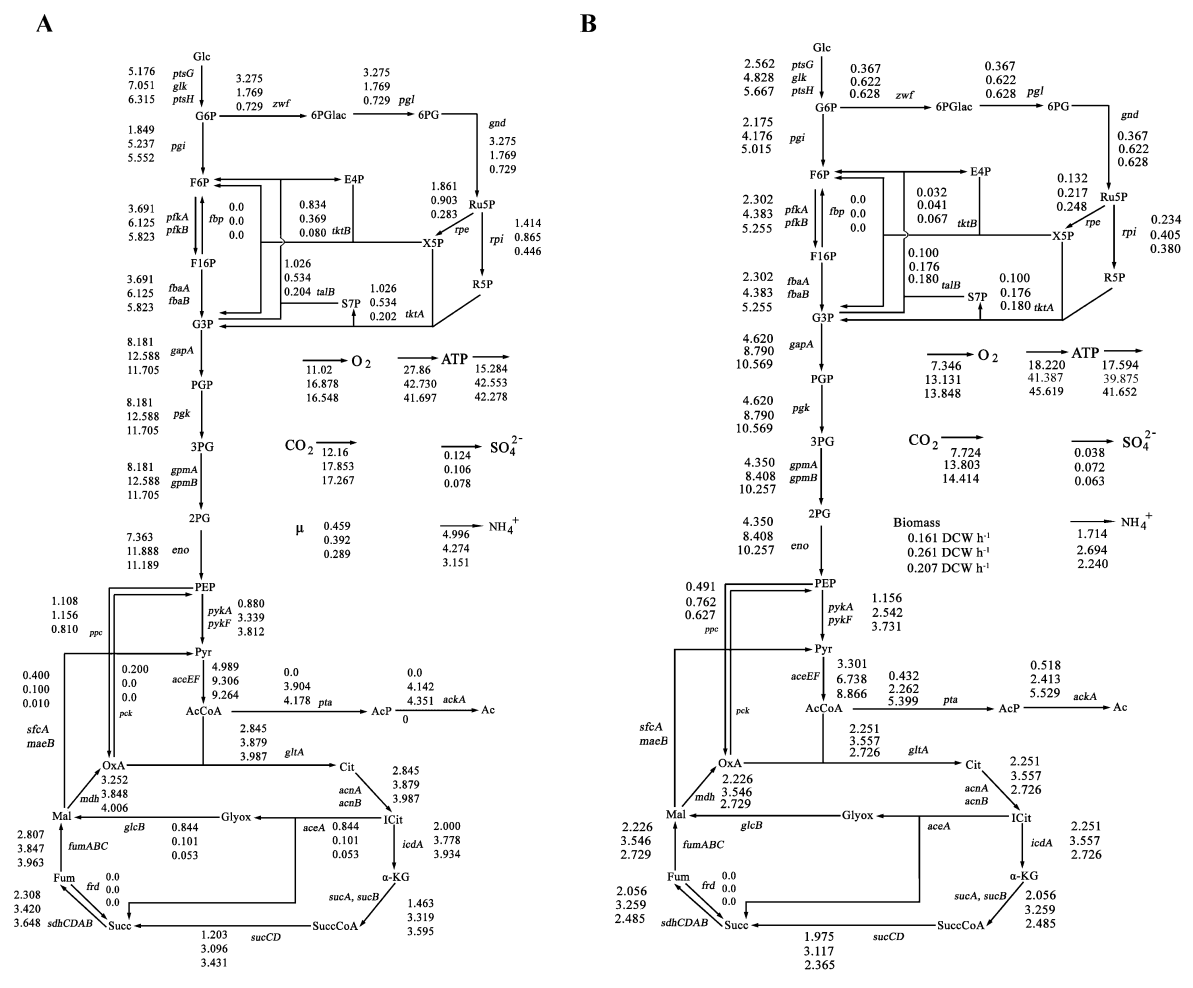
Metabolic flux results of *E. coli *BL21, BL21/pOri1 and BL21/pOri2 in chemostat system with the early stationary phase BL21 (0.46 h^-1^), BL21/pOri1 (0.39 h^-1^) and BL21/pOri2 (0.29 h^-1^) (A), and with the growth rate of 0.2 h^-1 ^(B). The flux results (BL21: Up; BL21/pOri1: Middle; BL21/pOri2: Down) are shown in panels A and B. Abbreviations: Glc: glucose; G6P, glucose-6-phosphate; F6P, fructose-6-phosphate; F16P, Fructose-1,6-bisphosphat; G3P, Glyceraldehyde-3-phosphate; PGP, 1–3-diphosphoglycerate; 3PG, 3-Phosphoglycerate; 2PG, 2-Phosphoglycerate PEP, phosphoenolpyruvate; Pyr, pyruvate; 6PGlac, 6-Phospho-Gluconolactone; 6PG, 6-Phospho-Gluconate; Ru5P, ribulose-5-phosphate; X5P, xylulose-5-phosphate; R5P, ribose-5-phosphate; S7P, sedoheptulose-7-phosphate; E4P, Erythrose 4-phosphate; AcCoA, Acetyl coenzyme A; AcP, Acetyl-P; Ac, acetate; Cit, Citrate; ICit, Iso-Citrate; α-KG, α-ketoglutarate; SuccCoA, Succinyl-Coenzym-A; Succ, succinate; Fum, fumarate; Mal, malate; OxA oxaloacetate.

The final distributions of metabolic fluxes in strains BL21, BL21/pOri1 and BL21/pOri2 were determined with FluxAnalyzer 5.1 software [26]. The metabolic fluxes of BL21, BL21/pOri1, BL21/pOri2 at the early stationary phase are shown in Fig. 3A, and those at the same growth rate are shown in Fig. 3B.

Glycolysis starts from glucose, and leads to pyruvate as its final product [27]. From the metabolic flux analysis, the levels of glycolytic intermediates, such as G6P, F6P, F1,6P, G3P, PGP, 3PG, 2PG, PEP and Pyr were significantly increased in the BL21/pOri1 and BL21/pOri2 relative to BL21 during the early stationary phase (Fig. 3A).

A quantitative description of the relative fluxes is important in the analysis of the PP pathway and glycolysis pathway. During the early stationary phase, the fluxes of 6PGlac in BL21, BL21/pOri1 and BL21/pOri2 were determined as 3.275, 1.769 and 0.729 mmol g^-1 ^h^-1^, respectively (Fig. 3A). The 6PGlac flux of BL21/pOri1 decreased to 54.01 % of that estimated in BL21, and the 6PGlac flux of BL21/pOri2 decreased to 41.20 % of that estimated in BL21/pOri1. During the early stationary phase, leveles of PP pathway intermediates, such as 6PGlac, 6PG, Ru5P, X5P, R5P, E4P, S7P were significantly decreased in BL21/pOri1 and BL21/pOri2 relative to BL21 (Fig. 3A). As for the BL21/pOri2 strain, the PP pathway represented 11.54% of the glycolysis pathway flux (6PGlac from G6P), versus 25.09% for BL21/pOri1 metabolism, while the PP flux for BL21 was determined to be 63.27% of the glycolysis flux (Fig 2A). The flux ratio of F6P from G6P was increased in BL21/pOri1 and BL21/pOri2 relative to BL21, however, the flux ratio of F6P from X5P or X7P was decreased in plasmid-carrying *E. coli *relative to plasmid-free *E. coli *(Fig 2A). The flux ratios of OxA from PEP, PEP from OxA, Pyr from Mal, and Mal from Glyox were decreased in BL21/pOri2 or BL21/pOri1 relative to in BL21 (Fig 2A).

During the early stationary phase, the flux ratio of Mal from Glyox derived from the glyoxylate cycle was significantly decreased in BL21/pOri1 or BL21/pOri2 relative to BL21 (Fig. 3A), and the flux ratio of ICit toward α-KG was increased in BL21/pOri1 and BL21/pOri2 in comparison to ICit toward α-KG in BL21 (Fig. 3A). The α-KG contributed an important flux for the synthesis of amino acid and nucleotides.

The BL21/pOri1 revealed an increase in Cit in TCA cycle flux by 36.9% (from 2.845 mmol g^-1 ^h^-1 ^in BL21 to 3.879 mmol g^-1 ^h^-1 ^in BL21/pOri1) (Fig. 3A). However the presence of BL21/pOri2 had a minor effect on this paramenter relative to BL21/pOri1 (Fig. 3A). Thus, *E. coli *BL21 carrying a high copy number plasmid pOri2 did not significantly influence the TCA cycle relative to the host bearing the low copy number plasmid pOri1 (Fig. 3A).

It was demonstrated previously that malic enzyme plays a central role in increasing levels of lipids [28]. Malic enzyme catalyses converting Mal to Pyr, and it is NADP^+ ^dependent [29]. The presence of flux from Mal to Pyr can be determined by comparison of labeling of the C-1 and C-2 carbon atoms of Pyr with those of PEP [30]. In our experiments, during the early stationary phase, the metabolic flux from Mal to Pyr decreased significantly from 0.4 mmol g^-1 ^h^-1 ^in BL21, to 0.01 mmol g^-1 ^h^-1 ^in BL21/pOri2. However, similar malic enzyme activities were found in BL21/pOri2, BL21/pOri1 and BL21 (Table 3). This can be explained by a significant decrease in the glyoxyate cycle fluxes. The flux from ICit to Glyox decreased from 0.844 mmol g^-1 ^h^-1 ^in BL21 to 0.053 mmol g^-1 ^h^-1 ^in BL21/pOri2 (Fig. 3A). It has been found that malic enzyme activity was high under conditions favourable for lipid accumulation [29]. Our results show that BL21 carrying high copy number plasmid pOri2 did not reveal an increase in lipid accumulation relative to BL21 carrying a low copy number plasmid pOri1 (Table 5).

From the metabolic flux analysis, during the early stationary phase, the ATP production and expenditure in BL21 were 27.86 mmol g^-1 ^h^-1^, and 15.28 mmol g^-1 ^h^-1^, respectively (Fig. 3A). Thus, it appears that ATP should not be a limiting factor in BL21. However, in BL21/pOri2, the ATP production and expenditure were 41.55 mmol g^-1 ^h^-1^and 42.28 mmol g^-1 ^h^-1^, respectively (Fig. 3A). Hence, ATP was one of the limiting factors in BL21 carrying a high copy number plasmid pOri2. From the metabolic flux analysis during the early stationary phase, the uptake fluxes of SO_4 _^2- ^and NH_4 _^+ ^were decreased in BL21/pOri1 and BL21/pOri2 relative to BL21 (Fig. 3A), possibly because *E. coli *cells carrying plasmid revealed lower growth rates than plamid-free *E. coli *cells.

From our experimental results, it appears that during the early stationary phase, the PP pathway was one of the important limiting factors of *E. coli *BL21 containing a high copy number plasmid, while the TCA pathway was not a limiting factor for *E. coli *BL21 containing a high copy number plasmid relative to a low copy number plasmid.

To determine the effects of growth rates on the metabolic flux analysis, we continuously cultured BL21, BL21/pOri1, BL21/pOri2 under the same growth rates as 0.2 h^-1^. The metabolic flux analysis results are shown in Fig. 3B. Surprisingly, TCA flux in the high copy number plasmid carrying *E. coli *BL21/pOri2 was lower than in the case of low copy number plasmid BL21/pOri1. Thus, TCA was one of limiting factors for plasmid DNA replication at the relatively low growth rate 0.2 h^-1 ^(Fig. 3B). Several key fluxes in the PP pathway in the high copy number plasmid-carrying *E. coli *BL21/pOri1 were higher than in the low copy number plasmid-carrying *E. coli *BL21/pOri2 (Fig. 3B) except the flux from Ru5P to R5P. These results indicate that the fluxes from Ru5P to R5P might be limiting factors for ColE1-like plasmid DNA replication. Moreover, ATP was not a limiting factor for plasmid DNA replication when BL21/pOri2 was cultured at relatively lower growth rate 0.2 h^-1 ^(Fig. 3B).

In summary, from the metabolic flux analysis of *E. coli *BL21/pOri1, BL21/pOri2 at the early stationary phase and at the same growth rate, presented in the report, one may conclude that the flux from Ru5P to R5P is the important limiting factor for ColE1-like plasmid DNA replication.

### Effects of plasmids on E. coli gene expression

Transcription profiles in BL21, BL21/pOri1 and BL21/pOri2 were analysed using DNA microarray technology. The strains were cultured in a chemostat and harvested after the cultivation reached the steady status. Total RNA was isolated and transcripts were reverse-transcribed into cDNAs. The RNAs from the BL21, BL21/pOri1, BL21/pOri2 were labelled with biotin.

The labelled cDNAs were mixed and then hybridized on the microarray slides. Experiments were repeated four times. Expression datasets have been deposited in the Gene Expression Omnibus database [GEO:GSE5239].

Over 3100 genes were successfully detected in the DNA microarray experiments. Most of them revealed no significant differences between plasmid-carrying *E. coli *and plasmid-free *E. coli*. Table 7 shows the selected results of DNA microarray experiments. In the carbon catabolism, there were number of genes, which were up-regulated significantly, suggesting that these groups of genes were helpful for maintaining plasmid DNA in *E. coli*.

**Table 7:** DNA microarray analysis of expression of selected genes in *E. coli *BL21, BL21/pOri1 BL21/pOri2 grown in the chemostat system at different growth rates.

Gene	Ratio of gene transcripts^a^			
	
	A	B	C	D
*rpiA*	0.59	0.03	0.45	0.04
*tktB*	0.11	0.13	0.07	0.19
*sfcA*	0.25	0.24	0.19	0.21
*gnd*	0.30	0.31	0.26	0.48
*maeB*	0.31	0.30	0.26	0.47
*talB*	0.31	0.32	0.35	0.40
*rpe*	0.41	0.40	0.42	0.62
*zwf*	0.50	0.52	0.59	0.55
*aceB*	0.51	0.48	0.55	0.69
*aceK*	0.63	0.61	0.51	0.92
*tktA*	0.61	0.63	0.55	0.99
*sucD*	2.80	3.10	2.01	3.25
*pykF*	2.80	3.10	1.83	3.17
*sdhC*	2.90	3.50	3.19	4.66
*eno*	2.90	2.50	3.02	3.47
*sucC*	3.02	3.12	3.25	3.41
*pta*	3.00	3.60	3.34	5.11
*sucB*	3.10	3.40	3.59	3.10
*icdA*	3.20	5.10	2.45	6.23
*gltA*	3.20	4.10	3.62	5.60
*gapA*	3.60	4.10	4.01	6.19
*fbaA*	3.60	3.80	2.38	5.41
*acnB*	3.60	4.60	3.80	4.36
*sucA*	3.60	3.90	3.93	3.76
*ptsG*	3.80	4.10	4.48	6.50
*ackA*	4.80	5.40	4.83	7.92
*fumB*	4.90	5.10	3.45	8.08
*fumA*	5.20	6.40	5.83	9.30
*acnA*	5.90	6.70	6.77	9.96
*fumC*	6.60	6.50	4.38	10.30

^a ^The results are average values from 4 experiments. Abbreviations: A, ratio of BL21/pOri1 (μ = 0.39) to BL21 (μ = 0.46); B, ratio of BL21/pOri2 (μ = 0.29) to BL21 (μ = 0.46); C, ratio of BL21/pOri1 (μ = 0.20) to BL21 (μ = 0.20); D, ratio of BL21/pOri2 (μ = 0.20) to BL21 (μ = 0.20).

The expression levels of many carbon transport genes were also affected in BL21/pOri2 and BL21/pOri1 comparing with BL21. Expression of genes involved in glucose transport (*ptsG*) was increased significantly during the early stationary phase and the same growth status.

Genes involved in glycolysis (*fba*, *gapA*, *eno*, *pykF*) were significantly up-regulated in BL21/pOri2 and BL21/pOri1 relative to BL21, however the PP pathway genes (*zwf *and *gnd*) were significantly down-regulated in early stationary phase growing *E. coli *cells and at the same growth rate 0.2 h^-1 ^(Table 7). *pta *and *ackA*, genes responsible for the acetate metabolism [31,32], were upregulated in BL21/pOri1 and BL21/pOri2 strains (Table 7).

Most of the tricarboxylic acid cycle genes were up-regulated (including: *gltA*, *acnA*, *acnB*, *icdA*, *sucA, sucB, sucC, sucD*, *sdhC*, *fumA*, *fumB*, *fumC*) in BL21/pOri1 and BL21/pOri2 strains relative to the plasmid-free strain (Table 7).

The glyoxylate shunt genes, *aceB *and *aceK*, showed down-regulation in plasmid-bearing cells (Table 7). These results correlate with the enhanced TCA cycle pathway and an impaired glyoxylate pathway [33]. The *sfcA *and *maeB *genes, which are known to be the main gluconeogenic pathway genes in *E. coli*, were also down-regulated.

Among the genes involved in the cellular structure, DNA replication, transcription, and translation, only relatively few genes were slightly up-regulated or down-regulated in pleasmid-bearing cells.

Our results indicated that transcription of most of genes did not change significantly in BL21/pOri1 relative to BL21/pOri2. However, expression of the *rpiA *gene, encoding RPI enzyme, was significantly decreased in BL21/pOri2 relative to BL21/pOri1.

In summary, when considering enzyme activity analysis, metabolic flux analysis and DNA microarray results, the *rpiA *gene was selected as the key gene, which might affect biology of ColE1-like plasmids.

### Effects of the rpiA gene on ColE1-like plasmid copy number

To determine the role of the *rpiA *gene, a plasmid was constructed to overexpress this gene. This plasmid, pETrpiA, was introduced into BL21/pOri2 and BL21/pOri1, and two constructed strains BL21/pOri2/pETrpiA and BL21/pOri1/pETrpiA were used for analysis of effects of *rpiA *gene on the plasmid DNA copy number. During IPTG induction, BL21, BL21/pOri1, BL21/pOri2, BL21/pOri1/pETrpiA, BL21/pOri2/pETrpiA were cultured with the following growth rates: BL21, 0.46 h^-1^; BL21/pOri1,0.39 h^-1^; BL21/pOri2, 0.29 h^-1^; BL21/pOri1/pETrpiA, 0.39 h^-1^; or BL21/pOri2/pETrpiA, 0.29 h^-1^. Then, plasmid DNA copy number of pOri2 and pOri1 were determined by the real-time PCR method.

**Figure 4:**
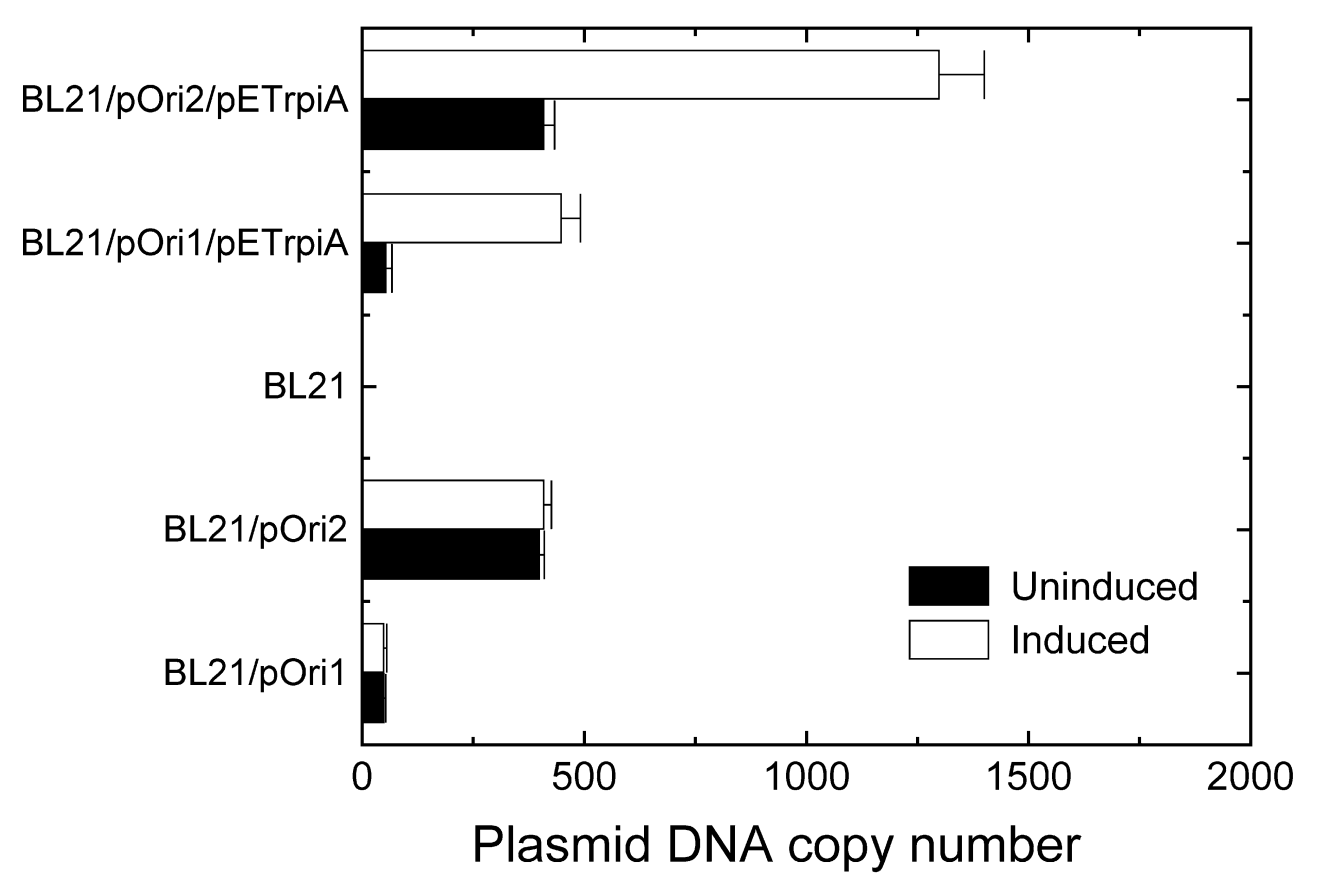
Plasmid pOri1 and pOri2 copy number in BL21, with or without the presence of pETrpiA plasmid, uninduced (■) or induced (□) wiith IPTG. Plasmid copy number was determined using a real-time PCR method.

A significantly increased pOri2 and pOri1 plasmid DNA copy number was found after induction of *rpiA *expression with 1 mM IPTG for 1 h (Fig. 4). These results suggest that in *E. coli *carrying a high copy number plasmid pOri2, the RPI enzyme is deprived, which in turn causes a decrease of the plasmid copy number. Thus, overproduction of RPI significantly increased the plasmid DNA copy number in *E. coli*.

## Discussion

Plasmids, as non-essential DNA molecules, require additional intracellular materials and energy to exist in host cell, thus usually reducing the growth rate of a bacterial culture [8,27,34]. Plasmid DNA has been considered as a molecular parasite [8] or a minimal genome [35]. However there is still a lack of a global analysis of the effects of plasmid DNA on the metabolism of *E. coli *[6].

Cultivation conditions affect plasmid DNA content significantly [36,37]. Andersson et al. [38] used the batch culture to analyse the effect of plasmid DNA on the metabolism of *E. coli*, and Klemperer et al. [39] analysed the requirements of minimal nutritional conditions of *E. coli *in batch culture with or without plasmid DNA. However, in the batch cultivation, plasmid DNA content might be significantly different between different growth status. Therefore, on the basis of those studies, it is difficult to analyse the effects of plasmid DNA on the metabolism of *E. coli*. We considered that the use of chemostat cultivation system is appropriate for plasmid DNA metabolism analysis.

A direct comparison between plasmid-bearing and plasmid-free *E. coli *may be inappropriate to find the metabolic limiting factors of plasmid DNA replication [8]. Rozkov et al. [6] have found that significant differences between a plasmid-free *E. coli *and the same host bearing a plasmid were due to expression of the kanamycin phosphotransferase gene (used as a plasmid marker). Thus, it appears that to analyse the metabolic limiting factors of plasmid copy number, a comparison of plasmid-containing with plasmid-free cells may be inaccurate.

Another methodological problem arises from the finding that growth rates affect the cell biomass significantly [40], and biomass ingredients have direct effects on the metabolic flux [41]. The presence of plasmids has significant effects on the ingredient of biomass, thus it is not reasonable to assume that the biomass is constant, which makes problems in interpretation of results to analyse the effects of plasmids on the metabolism of host cells.

To avoid all these problems, we constructed two different plasmids, derived from ColE1. The first plasmid (pOri1) contains one ColE1 replication origin, and the second plasmid (pOri2) contains two ColE1 plasmid DNA replication origins. Moreover, we used a stable culture status to analyse the effects of plasmid DNA on the metabolism of *E. coli*. Plasmid DNA copy number was significantly increased when two origin regions were present. We assumed that comparison of BL21/pOri1 and BL21/pOri2 should be accurate to analyse the effects of plasmid DNA on the metabolism of *E. coli*. Moreover, we have used the ^13^C-labelling experiments, DNA microarray and enzyme activity analysis to explore the metabolic differences. The ^13^C-labelling experimental results provided a large body of data to calculate the intracellular fluxes, and the isotopic enrichment in the intracellular metabolite pools. This technique, in a combination with DNA microarray and enzyme activity analysis, is a powerful method for screening of metabolic nodes or bottlenecks for plasmid DNA metabolism.

In our experiments, during early stationary phase, ColE1-derived plasmids pOri1 and pOri2 have significant effects on the metabolism of *E. coli*. TCA cycle, respiration rate and acetate yield were significantly increased in BL21/pOri1 and BL21/pOri2 strains. Also, the PP pathway flux was significantly decreased, and the ATP expenditure was increased in BL21/pOri2 significantly more than in BL21/pOri1. These results suggest potential sources of metabolic burden during plasmid DNA replication in the early stationary phase.

In experiments with the controlled cultivation growth rate at 0.2 h^-1^, the effects of ColE1-like plasmids pOri1 and pOri2 on the *E. coli *BL21 metabolism were significantly different relative to experimental results obtained from the experiments with early stationary phase. Our results show that PP pathway flux was generally increased, except the flux from Ru5P to R5P, in BL21/pOri2 relative to BL21/pOri1. From the metabolic flux analytical results obtained from the cultivation at the early stationary phase and at the same growth rate, we conclude that the flux from Ru5P to R5P is important for plasmid DNA replication, while TCA cycle, ATP or glucose uptake are not the limiting factors. To confirm these results, we performed enzyme activity analysis and mRNA expression profiles analysis.

The enzyme activity analysis was performed in bacterial cultures kept in the early stationary phase and the same growth rate. The glycolysis enzyme activities and most TCA enzyme activities were increased in BL21/pOri1 and BL21/pOri2 relative to BL21, while the enzyme activities of the PP pathway were significantly decreased. Moreover, the RPI enzyme activity was decreased significantly.

The gene expression profiles, obtained from DNA microarray experiments, were consistent with the results for early stationary phase and at the same growth rate. The gene expression profiles for BL21/pOri1, BL21/pOri2 and plasmid-free cells agree with the enzyme activity analysis. In BL21/pOri2 cultures, the phosphotransferase system for glucose uptake and glycolytic genes were up-regulated in comparison to those in BL21 cultures. On the other hand, genes involved in acetate secretion and the TCA cycle were up-regulated in BL21/pOri2 and BL21/pOri1, whereas the genes involved in the glyoxylate cycle and gluconeogenesis were down-regulated. The most striking difference between BL21/pOri2 and BL21/pOri1 was the regulation of PP pathway genes. These genes are either essential or carry significant metabolic flux for nucleotides' synthesis. Expression of the *rpiA *gene was significantly decreased in BL21/pOri2. From the metabolic flux analysis, enzyme activity analysis and mRNA expression profiles, we concluded that the *rpiA *gene is one of the important limiting factors for plasmid DNA replication.

The PP pathway has fewer enzymes than glycolysis, however, this pathway is more complicated [42]. It is involved in the generation of NADPH for biosynthesis, recruiting essential metabolites for nucleic acids, amino acids and vitamins, and the generation of ingredients of the cell lipopolysaccharide layer [43]. Amino acids and nucleotides are important for plasmid DNA replication. Moreover, during plasmid DNA replication, especially when a plasmid occurs at high copy number, an extra synthesis of nucleotides is needed. Thus, it is possible that the carbon flux, which is driven through the oxidative branch of the PP pathway, is not enough to handle the cell metabolic needs [44], particularly when the growth rate is decreased. Furthermore, the PP pathway is the only pathway that allows *E. coli *to use some sugars, such as D-xylose, D-ribose, or L-arabinose [45,46].

RPI is the enzyme that converts Ru5P into R5P, and this enzyme activity decreases the flux into X5P. This flux is not redirected into the Embden-Meyerhof (EM) pathway, and a large part of the flux into Ru5P is used for synthesis of nucleotides. RPI plays a key role in ribose 5-phosphate metabolism [47]. *E. coli *has two recognisable ribose-5-phosphate isomerase: a constitutive ribose-5-phosphate isomerase A and an inducible ribose-5-phosphate isomerase B, the latter being present in distinguishable amounts only when cells grow on D-ribose or ribose-5-P-delivering compounds, such as adenosine or uridine, as the sole carbon source [45,46,48]. It has been demonstrated that adenosine and uridine can be used to increase plamsid DNA copy number [44]. Thus, we suppose that inducible ribose-5-phosphate isomerase B can be used to increase ColE1-like plasmid DNA copy number. Although ribose-5-phosphate isomerase A could not be induced by D-ribose or ribose-5-P-delivering compounds, an expression of the gene coding for ribose-5-phosphate isomerase A, using a plasmid system, should lead to a significant increase in the total activity of this enzyme in the cell. In fact, Hove-Jensen and Maigaard found that a plasmid containing the *rpiA *gene can significantly (about 40-fold) increase the ribose-5-phosphate isomerase A activity in *E. coli *[47]. These results show that the ribose-5-phosphate isomerase A activity can be increased when the protein overproduction system is employed. Although Hove-Jensen and Maigaard did not analyze the effects of ribose-5-phosphate isomerase A activity on plasmid DNA replication [47], here, we found that the overproduced ribose-5-phosphate isomerase A can cause a significant increase in the ColE1 plasmid DNA copy number.

Considering all the data mentioned above, we have tested ColE1-like plasmid DNA copy number in *E. coli *cells overproducing the ribose-5-phosphate isomerase A. In these cells, copy number of plasmids pOri1 and pOri2 was significantly increased, indicating that RPI may be a limiting factor for ColE1-like plasmid DNA replication, indeed.

Our results have also potential biotechnological applications. Namely, modification of the PP pathway by enhancement of the *rpiA *gene expression can significantly increase ColE1-like plasmid DNA copy number in *E. coli*. In the light of the role of plasmids in gene therapy and development of DNA vaccines, such improvement may be considerable. Increased plasmid copy number may also lead to more effective expression of recombinant genes coding for desrired proteins. In fact, it was demonstrated previously that the PP pathway has an important limitation in the metabolism of *E. coli *[49], namely, the growth rate and recombinant protein expression were increased in *E. coli *cells overproducing the glucose-6-phosphate dehydrogenase.

## Materials and methods

### Bacterial strains, plasmid construction and cultivation conditions

*E. coli *strain BL21(DE3) (Invitrogen, CA) and ColE1-derived plasmids pOri1, pOri2 were used. Fig. 1 shows the constructions of pOri1 and pOri2. pOri1 plasmid contains a single ColE1 replication origin and an ampicillin-resistance gene, while pOri2 plasmid contains two ColE1 replication origins and an ampicillin-resistance gene. The designed forward primer: 5' GCA ATC CAA ATG GGA TTG CTA GGA 3' and reverse primer: 5' CAT CGG TAT CAT TAC CCC ATG AAC 3' were used to amplify the replication origin of ColE1 plasmid [GenBank:NC001371] with AccuPrime *Pfx *DNA polymerase (Invitrogen, CA). The following two-step PCR reactions were performed: one cycle at 95°C for 2 min, 35 cycles at 95°C for 15 s, 68°C for 1 min. Finally, PCR products were kept at 4°C after cycling. Another PCR reaction was used to amplify an ampicillin-resistance gene from pUC18 plasmid (Invitrogen, CA) with the forward primer: 5' GAG TAA ACT TGG TCT GAC AGT 3' and reverse primer: 5' GGT TAA TGT CAT GAT AAT AAT 3'. The blunt-end PCR product of ColE1 replication origin region was linked with 5' phosphorylated blunt-end PCR product of an ampicillin-resistance gene to construct plasmid pOri1. Orientation of the insert was confirmed by DNA sequencing. Another PCR reaction was performed using the forward primer: 5' GCA ATC CAA ATG GGA TTG CTA GGA 3', and reverse primer: 5' GGT TAA TGT CAT GAT AAT AAT 3' to amplify the whole pOri1 plasmid with AccuPrime *Pfx *DNA polymerase. The amplified PCR product was linked with 5' phosphorylated PCR product of the ColE1 replication origin fragment to construct plasmid pOri2. Thus, plasmid pOri2 contains two ColE1 replication origins. Both pOri1 (2,811 bp) and pOri2 (4,575 bp) were used to transform BL21 cells.

Single clones were selected from the LB plates containing 100 μg ml^-1^ampicillin, and cultured in LB medium for 12 h. Then 5 ml culture broth was inoculated into 125 ml of a fresh LB medium including 100 μg ml^-1^ampicillin for another 10–15 h. Finally, *E. coli *cells were washed with sterilized PBS buffer (phosphate buffered saline, pH7.2) before inoculation into the fermentor.

All cultures were carried out in a 5 l fermentor BIOSTAT B-DCU (Sartorius BBI Systems Inc. Melsungen) at controlled temperature 37°C, pH 7.0, and dissolved oxygen tension 30%. The culture volume was 2 l. All reagents were purchased from Sigma-Aldrich, Inc. The synthetic culture medium consisted of (values in g l^-1^): 8, glucose; 14.6, K_2_HPO_4_; 3.6, NaH_2_PO_4 _· H_2_O; 2.68, (NH_4_)_2_SO_4_; 2, Na_2_SO_4_; 1, MgSO_4_; 1, Na-citrate; 0.5, NH_4_Cl; 2 ml of 10 mg l^-1 ^of thiamine; and 3 ml of trace element solution (in g l^-1^): 20.0, Na-EDTA; 15.0, FeCl_3 _· 6H_2_O; 0.5, CaCl_2 _· 2H_2_O; 0.2, ZnSO_4 _· 7H_2_O; 0.2, CoCl_2 _· 6H_2_O; 0.2, CuSO_4 _· 5H2O; 0.2, MnSO_4 _· 4H_2_O. The chemostat culture was performed by addition of feed medium (the composition was the same as the culture medium). The continuous culture reached steady state growth after five residence times with the specific growth rate; no significant plasmid DNA lose (as estimated by determining a fraction of amplicillin-resistant bacteria) was found during the cultivation. Labelling experiments were started after the chemostat culture reached a steady state, then the feed medium containing 8 g l^-1 ^of unlabelled glucose was replaced with the medium containing 1.8 g l^-1 ^[U-^13^C]glucose and 6.2 g l^-1 ^of natural glucose, or 8 g l^-1^of [1-^13^C] glucose. The samples were collected after one residence time when the substrate was changed to the labelled glucose.

### Determination of bacterial ingredients

DNA content was measured by the diphenylamine reagent method with calf thymus DNA as a standard [50]. Amounts of genomic DNA were obtained by subtracting plasmid DNA from total DNA amount. RNA level was determined by the Schmidt-Tannhauser method [51].

For the analysis of free amino acids, freeze-dried biomass was suspended in distilled water. The biomass was boiled in water for 15 min. The suspension was centrifuged at 10,000 *g *for 10 min, and the free amino acids in the supernatant were analysed using high-performance liquid chromatography (HPLC) series 200 LC Plus UV/Vis System (PerkinElmer, MA). The total pool of amino acids was analysed by HPLC after *E. coli *samples were hydrolyzed with 6 M HCl for 24 h at 110°C. Values for amino acids derived from proteins were obtained by subtracting the values of free amino acid from the total amino acid amout.

For the analysis of total nitrogen content in *E. coli*, samples were hydrolyzed with 6 M HCl for 24 h at 110°C. The nitrogen content originating from protein in *E. coli *was calculated using the method of subtracting the nitrogen contents in RNA, DNA, and free amino acids from the total nitrogen content measured by the Kjeldahl method [52]. The protein content was determined according to nitrogen content originating from proteins in *E. coli*. Lipids were extracted with chloroform and methanol. The lipid content was determined by gas chromatography (GC) analysis with Clarus 500 Gas Chromatograph (PerkinElmer, MA) [53]. Glycogen contant was determined by the method described by Dauvillee et al. [54], peptidoglycan was determined by the method described by Chandrakala et al. [55] and LPS was determined by the method described by Boman and Monner [56].

### Analysis of growth and enzyme activity

Dry cellular weight (DCW) from the cultures was monitored by optical density at 600 nm (OD_600_) and converted to DCW based on the determined OD-to-DCW correlations. Glucose concentration was determined using glucose assay kit (Sigma). Acetate concentrations in the culture broth were measured by HPLC. CO_2 _production and O_2 _consumption were measured by passing the outgoing gas from the fermentor through a Servomex CO_2 _and O_2 _analysers (Servomex Company Inc. TX), respectively [57]. Specific enzymes, which are located at the main branch points of the central metabolic pathways, were analyzed in crude cell extracts from *E. coli *BL21, BL21/pOri1 or BL21/pOri2. Enzyme activity was expressed as the amount of enzyme required to convert 1 μmol substrate into specific product per minute per milligram of protein. *E. coli *cells were harvested from 500 ml culture broth by centrifugation at 10,000 *g *for 10 min, washed twice with TE buffer (100 mM Tris-HCl, pH 7.0, 0.1 mM EDTA), then resuspended in 50 ml of TE buffer and disrupted in an ultrasonicator. The cellular debris was removed by centrifugation, and the resulting crude cell extracts were immediately used for determination of specific enzyme activities or stored at -70°C. Protein concentrations were estimated using a QuantiPro high sensitivity protein assay kit (Sigma). Enzyme activities were measured spectrophotometrically at 37°C in a DU Series 500 UV/Vis Spectrophotometer (Beckman, CA). Reaction mixture and substrate were added into a cuvette with a 1 cm light path, and reactions were initiated by adding the cell extract or substrate to give the final volume of 1 ml. The same amount of protein was added to compare the enzyme activities of different samples, and each measurement was performed in triplicate. Activities of all selected enzymes were measured using the already published methods, as follows: Hexokinase (HEK) (EC 2.7.1.1) [58]; Glucose-6-phosphate isomerase (GPI) (EC 5.3.1.9) [59]; 6-phosphofructosekinase (PFK) (EC 2.7.1.11) [60]; Fructose-1,6-bisphosphatase (FBP) (EC 4.1.2.13) [60]; Fructose-bisphosphate aldolase (FBA) (EC 4.1.2.13) [58]; Glyceraldehyde-3-phosphate dehydrogenase (GPD) (EC 1.2.1.12) [61]; Triose phosphate isomerase (TPI) (EC 5.3.1.1) [62]; Phosphoenolpyruvate carboxylase (PPC) (EC 4.1.1.31) [63]; Pyruvate kinase (PK) (EC 2.7.1.40) [62]; Glucose-6-phosphate dehydrogenase (GPDH) (EC 1.1.1.49) [61]; 6-Phosphogluconolactonase (PGL) (EC 3.1.1.31) [64]; Phosphogluconate dehydrogenase (PGD) (EC 1.1.1.44) [61]; Ribulose-phosphate 3-epimerase (RPE) (EC 5.1.3.1) [65]; Ribose-5-phosphate isomerase (RPI) (EC 5.3.1.6) [47]; Transketolase (TK) (EC 2.2.1.1) [66]; Transaldolase (TA) (EC 2.2.1.2) [67]; Citrate synthase (CS) (EC 2.3.3.1) [68]; NADP^+^-specific isocitrate dehydrogenase (ICD) (EC 1.1.1.42) [69]; Isocitrate lyase (ICL) (EC 4.1.3.1) [63]; Aconitate hydratase (AH) (EC 4.2.1.3) [70]; Oxoglutarate dehydrogenase (OGD) (EC 1.2.4.2) [71]; Succinic semialdehyde dehydrogenase (SD) (EC 1.2.1.16) [63]; Fumarate hydratase (FH) (EC 4.2.1.2) [72]; Malate dehydrogenase (MD) (EC 1.1.1.37) [73]; Malic enzyme (MAC) (EC 1.1.1.40) [63]; Phosphoenolpyruvate carboxykinase (PCK) (EC 4.1.1.38) [63].

### Analysis of plasmid DNA copy number with real-time PCR method

10^9 ^*E. coli *cells were harvested from the culture broth, and the pellets were resuspended with 250 μl TGE buffer (1M Tris-HCl, pH 8.0; 50 mM, glucose; 0.5 M EDTA, pH 8.0). Then, 250 μl of SDS-NaOH lysis buffer (0.2 M NaOH; 1% SDS) and 350 μl of 3 M potassium acetate (adjusted to pH 4.8 with acetic acid) were added. After incubated on ice for 10 min, the mixture was centrifuged at 12,000 *g *for 10 min, and the finial supernatants were used for real-time PCR analysis of the plasmid DNA concentration.

QuantiTect SYBR Green PCR kit (Qiagen) was used for determination of plasmid DNA concentration. The forward primer: 5' ATG AGT ATT CAA CAT TTC CGT GTC 3' and reverse primer: 5' CTT CCG GCT GGC TGG TTT ATT GCT 3' were used to amplify an ampicillin-resistance gene in pOri1 or pOri2 plasmid. The PCR reactions were performed in iQ5 real-time PCR detection system (Bio-rad, CA). The serial dilutions of pOri1 or pOri2 plasmid DNA solutions were used as standards for determination of plasmid DNA concentration in the cellular lysate.

Plasmid DNA copy number was determined employing the following equation: 6.02 × 10^23 ^× (*C *× *V*)/(*N *× *M *× 2 × BP), where C is the plasmid DNA concentration in the lyaste; V is the volume of cleared lysate; N is the total *E. coli *cell number for plasmid copy number determination, M is the formula 'molecular' weight of plasmid DNA, which was determined as described previously [44]. BP is the number of base pairs in plasmid DNA.

### Metabolic flux analysis with ^13^C-labeled glucose

To obtain ^13^C-labeled amino acids from a biomass, 2 ml of bacterial culture were harvested and centrifuged at 12,000 g, 4°C for 2 min. Cell pellets were washed with 20 mM Tris · HCl (pH 7.6) and hydrolyzed in 0.5 ml of 6 M HCl at 105°C for 24 h. Following liofilization drying the sample under vacuum, it was suspended in 1 ml of anhydrous ethanol. Then, the ethanol was evaporated in a vacuum at room temperature. 100 μl of acetonitrile and 100 μl of *N*-methyl-*N*-(*tert*-butyldimethylsilyl)trifluoroacetamide (MTBSTFA) were added to the dried sample. The reaction mixture was heated at 70°C for 30 min. After cooling to room temperature, the suspension was filtered through cotton. This solution was directly injected onto the GC column. The derived amino acids were firstly separated by the GC, then ionized, and subsequently fragmented in the MS. The fragments contained different subsets of the original carbon skeleton. The most common MS fragments of the N(O)-tert-butyldimethylsilyl amino acids were as follows: [M – 15]^+ ^(loss of CH_3_); [M – 57]^+ ^(loss of C(CH_3_)_3_); [M – 85]^+ ^(loss of C(CH_3_)_3_-CO); [M – 131]^+ ^(loss of OtBDMS); [M – 159]^+ ^(loss of COOtBDMS). The labelling patterns of free intracellular amino acids of *E. coli *were analysed by Clarus 500 GC mass spectrometer (PerkinElmer, MA).

For metabolic flux ratio analysis, mass spectra of the derivatized amino acids were corrected for the natural abundance of all stable isotopes. The metabolic flux ratio analysis was performed using the FiatFlux 1.0 software [25].

After determination of the metabolic flux ratio of *E. coli *with FiatFlux 1.0 RATIO package, the metabolic fluxes of *E. coli *were determined with FluxAnalyzer 5.1 software [26]. The reaction equations were designed according to the metabolic equations of *E. coli*, published previously [74]. The biomass was determined separately as protein, lipid, RNA, genomic DNA and plasmid DNA, LPS, peptidoglycan, and glycogen. The mathematical framework for flux estimation was predicted based on an initial set of fluxes, then calculated fluxes were compared with the measured values. This iterative procedure continued until the error between the calculated and measured values was below the set criterion (varying measurement and calculation was set as 0.01 for each reaction) with simple least squares methods.

### Transcriptome analysis

*E. coli *samples for RNA extraction were taken during fermentation. Cells were harvested by centrifugation at the cultivation temperature (37°C, 10,000 *g*, 1 min), separated from the supernatant, and rapidly frozen in dry ice. The samples were stored at -70°C until analysis. GeneChip *E. coli *Antisense Genome Array (Affymetrix, USA) chips were used for DNA microarray analysis. The array included more than 4,200 genes from *E. coli *K12. For transcriptome analysis, 15 μg of RNA from each sample was reverse-transcribed into cDNA with random primers. GeneChip DNA labelling reagent (Affymetrix, USA) was used to label the 3' ends of cDNA products. Finally, the labelled cDNA samples were hybridized to the cDNA array. Scanning was performed with a DNA Microarray Scanner BA (Agilent Technologies, Inc, CA).

### Cloning the rpiA gene from E. coli BL21

*E. coli *K12 genomic sequence [GenBank:NC000913] was used to design the primers, and the designed forward primer: 5' GGG GGA TCC GGA TGG GCG GCA CTT CAG TAT GTT 3' and reverse primer: 5' GGG CTC GAG TTA TTT CAC AAT GGT TTT GAC ACC GTC 3' were used for PCR reaction. The forward primer contains *Bam*HI cloning site, and the reverse primer contains the *Xho*I cloning site (underlined). PCR reaction was used to amplify the *rpiA *gene from BL21 genomic DNA. The amplified PCR product was digested with *Bam*HI and *Xho*I, respectively. The digested PCR products were inserted into the *Bam*HI- and *Xho*I-digested pET28a vector (bearing the pMB1 replication *origin*) by T4 DNA ligase. Finally, the ligation product was used to transform *E. coli *DH5α competent cells. Then, the clones were selected, and the constructed plamid pETrpiA was used to transform BL21, BL21/pOri1, and BL21/pOri2 strains.

To analyse the effects of pETrpiA on the plasmid pOri1 or pOri2 copy number in BL21, BL21/pOri1/pETrpiA, BL21/pOri2/pETrpiA, bacteria were cultured in 125 ml LB medium containing 100 μg ml^-1 ^ampicillin and 50 μg ml^-1 ^kanamycin. Before the *E. coli *cells were inoculated into 5 l fermentor with the working volume of 2 l, *E. coli *cells were washed with sterilized PBS buffer, then the synthetic culture medium was used for chemostat cultivation in 5 l fermentor. During the early stationary phase, BL21, BL21/pOri1/pETrpiA or BL21/pOri2/pETrpiA was induced with 1 mM IPTG. The final growth characteristics were analyzed.
